# Predictive echocardiographic factors of severe obstructive sleep apnea

**DOI:** 10.11604/pamj.2021.38.359.28470

**Published:** 2021-04-14

**Authors:** Imen Touil, Hassen Ibn Hadj Amor, Melek Kechida, Nadia Keskes Boudawara, Yosra Brahem, Soumaya Bouchareb, Mohamed Taha Hasnaoui, Leila Boussoffara, Jalel Knani

**Affiliations:** 1 Department of Pneumology, Taher Sfar Hospital, Mahdia, Tunisia,; 2 Department of Cardiology, Taher Sfar Hospital, Mahdia, Tunisia,; 3 Department of Internal Medicine, Fattouma Bourguiba Hospital, Monastir, Tunisia

**Keywords:** Sleep apnea, polygraphy, apnea index, echocardiography, right ventricular remodeling

## Abstract

**Introduction:**

obstructive sleep apnea (OSA) is a common chronic pulmonary disease, characterized by repetitive collapse of the upper respiratory airways, leading to oxygen desaturation. This condition is recognized to be associated with cardiovascular disease. Several studies have shown the effects of OSA on both geometry and cardiac function, with conflicting results. We aimed to investigate the relationship between echocardiographic abnormalities and the severity of OSA.

**Methods:**

this is a cross-sectional single center study including patients, without any cardiovascular or pulmonary comorbidities, with polygraphy proven OSA. All participants underwent a detailed transthoracic echocardiography (TTE).

**Results:**

a total of 93 patients were included in the study, with 62.2% (n=56) females. According to the apnea hypopnea index (AHI), patients were divided into two groups: mild to moderate OSA (5≤ AHI< 30/H) and severe OSA (AHI≥ 30/H). There were no differences in baseline characteristics between the two groups. The assessment of echocardiographic parameters demonstrated that severe OSA have a higher left ventricular end-systolic (LVES) (47.6±7.2 VS 46.2±4.7), left ventricular end-diastolic (LVED) (31.3±6.2 VS 28.9±4.5) diameters and interventricular septum (IVS) thickness (12.7±2.4 VS11.7±2.5) diameters rather than mild to moderate OSA without a significant difference between the two groups. Furthermore, severe OSA patients had lower mean value of left ventricular ejection fraction (LVEF) and fractional shortening (FS) equal to 62.1±9.7 and 32.5±6.3 respectively. The difference between the two groups was not statistically significant. However, a significant association was shown between severity of OSA and left ventricular (LV) diastolic dysfunction, right ventricular internal diameter (RVID) and systolic pulmonary artery pressure (sPAP), with p=0.05, p=0.05 and p= 0.03 respectively. The RVID was also independently associated to the severity of the OSA (aOR 1.33, 95%CI: 0.99-1.79; p=0.05).

**Conclusion:**

using bidimensional echocardiography showed a relationship between severe OSA and right ventricular parameters (diastolic dysfunction and RVID) and sPAP.

## Introduction

Obstructive sleep apnea (OSA) is a common sleep-related breathing disorder estimated to affect between 5% and 14% [1]. It is characterized by repetitive collapse of the upper respiratory airways, causing recurrent apneas (complete collapse) or hypopneas (incomplete collapse) intermittent and oxygen desaturations. Clinically, OSA is defined by the occurrence of daytime sleepiness, loud snoring, witnessed breathing interruptions, or awakenings due to gasping or choking in the presence of at least 5 obstructive respiratory events (apneas, hypopneas or respiratory effort related arousals) per hour of sleep [2]. Obstructive sleep apnea is associated with high mortality and morbidity rate [3]. In fact, various studies depict an association of this condition with hypertension, coronary artery disease, heart failure and cardiac arrhythmias [4]. The physiopathological interaction between OSA and cardiovascular disease is complex. The most accepted theory being a mismatch of myocardial oxygen supply and demand owing to repetitive nocturnal hypoxia and secondary sympathetic nervous system over activity [5]. Echocardiography is a noninvasive, low-cost and accurate tool for assessing alterations in cardiac structure and function. Previous studies have been performed to examine the effects of OSA on echocardiographic parameters [6]. Currently, there is sufficient evidence to prove that OSA is associated with subclinical cardiac structural or functional alterations. Although, the conventional echocardiography yields conflicting results regarding OSA severity. The aim of the current study was to investigate the relationship between echocardiographic abnormalities and the severity of OSA.

## Methods

**Study design and subjects:** this is a cross-sectional analytical study conducted at the Pneumology Department of Taher Sfar University Hospital. We included patients aged over 18 years referred to our sleep laboratory for OSA screening, between 2005 and 2017. Patients, newly diagnosed with OSA, but free of obstructive and/or restrictive respiratory or cardiovascular comorbidities (ischemic or valvular heart disease, hypertension, cardiomyopathy or chronic lung disease), were enrolled after obtaining detailed history and performing physical examination, routine blood examination, polygraphy and transthoracic echocardiography (TTE).

**Polygraphy:** all patients underwent an overnight polygraphy in the sleep laboratory using standard techniques. Electrocardiogram, chest and abdominal respiratory movements were made during the entire sleep duration. Oro-nasal airflow and arterial oxygen saturation (SaO_2_) were measured continuously. Sleep apnea refers to a reduction of ≥ 90% or complete obliteration in the respiratory airflow lasting ≥10s during sleep. Hypopnea was defined as a reduction ≥ 30% in the respiratory airflow lasting ≥10s and accompanied by a decrease ≥ 4% in oxygen saturation during sleep [7]. Obstructive sleep apnea was defined as an index apnea hypopnea (AHI) of ≥ 5 per hour during sleep in presence to the clinical symptoms.

**Grouping:** according to the AHI, OSA severity is classified into mild (5≤AHI<15), moderate (15≤AHI<30) and severe forms (AHI≥30 events/hour) [2]. Patients who met the aforementioned inclusion and exclusion criteria were recruited and divided into two groups: mild to moderate OSA and severe OSA.

**Echocardiography:** all the patients, recently diagnosed with OSA, underwent a comprehensive transthoracic echography examination according to the American society of echography guidelines [8]. The left ventricular ejection fraction (LVEF) was acquired using the modified biplane Simpson method from the apical 4 and 2-chamber views. Left ventricular end-systolic (LVESD) and end-diastolic LV diameters (LVEDD) were measured with M-mode in the parasternal long-axis view, while M-mode from long axis view was performed to measure the interventricular septum (IVS) thickness. Fractional shortening (FS) was calculated by measuring the percentage change in left ventricular diameter during systole. It was measured in parasternal log axis view using M-mode. The trans-mitral pulsed wave Doppler velocities were recorded from the apical 4 chamber view with a 2mm Doppler sample placed between the tips of the mitral leafets. Early (E) and late (A) wave velocities, E/A ratio and deceleration time were measured from the mitral inlow profle in order to evaluate the left ventricular diastolic function. The right ventricular internal diameter (RVID) was measured at the end-diastole from a right parasternal long-axis 4-chamber view. The right ventricular dilatation was defined by a mid-level diameter >35mm [9]. Transthoracic echocardiographic estimates of peak systolic pulmonary artery pressure (sPAP) are conventionally calculated from the maximal velocity of the tricuspid regurgitation. Pulmonary hypertension was defined by a sPAP ≥30 mmHg [10].

**Statistical analysis:** statistical analysis was performed with the statistical package for social sciences (SPSS) V.23 for Windows. Categorical variables were expressed in absolute values and proportions. Continuous variables were expressed as mean ± standard deviation. The relation of OSA severity with various echocardiographic parameters was assessed by Student's t-test or chi square test as deemed appropriate. Significant variables at the 15% level were introduced into multivariable analysis, binary logistic regression, in order to determine factors associated with severe OSA. Results were expressed as odd ratios (OR) with 95% confidence intervals (CI). A P value ≤ 0.05 was considered significant.

## Results

**General information:** a total of 93 patients fulfilled the eligibility criteria were included in the study. The mean age of the total sample was 48.5±9.7 years, with 62.2% (n=56) females. According to AHI, 29% (n=27) had mild OSA, 19.4% (n=18) moderate OSA and 51.6% (n=48) severe OSA. Mild and moderate OSA patients were comparable in terms of demographic and clinical parameters. The mean AHI was 9.67±3.01/h in mild group and 20.68±4.4/h in moderate group, with a statistically significant difference (p<10-3). Patients, enrolled in this study, were classified into 2 groups: mild/ moderate OSA group (48.4%, n= 45) and severe OSA group (51.6%, n= 48). The detailed demographic and clinical characteristics as well as polygraphic data of the 93 patients are presented in (Table 1). There were no differences in baseline characteristics including age, gender, rate of smoking, blood pressure and the body mass index (BMI) between the group of mild to moderate OSA patients and the group of severe OSA subjects. Comorbidities such as diabetes and dyslipidemia were similar between the two groups. However, the mean AHI in the first group was 14±6.5 and 48.2±17.3 in the second one with a statistically significant difference (P<10-3).

**Table 1:** demographic and clinical characteristics of the groups

	Study population (n=93)	Mild to moderate SOAS (n= 45)	Severe SOAS (n=48)	P
Mean age (years)	48.5±9.7	47.3±9.5 (30-75)	49.6±9.8 (30-74)	0.24
Female gender % (n)	60.2 (56)	64.4 (29)	56.3 (27)	0.42
Smoking % (n)	27.9 (26)	26.7 (12)	29.2 (14)	0.78
Systolic blood pressure (mmHg)	12.9±1.3	12.9±1.3	12.8±1.3	0.70
Diastolic blood pressure (mmHg)	7.5±0.9	7.7±0.9	7.4±0.8	0.09
BMI (Kg/m^2^)	34.1±6.6	33.7±6.5	34.5±6.7	0.57
Diabetes mellitus % (n)	16.1 (15)	17.8 (8)	14.9 (7)	0.71
Dyslipidemia % (n)	7.5 (7)	8.9 (4)	6.2 (3)	0.63
NC (cm)	43.4±7.9	42.9±8.4	44±7.2	0.65
Epworth scale	11.8±4.8	11.8±5.2	11.9±4.4	0.96
AHI (events/h)	30 [13-43]	14±6.5 (5-28)	48.2±17.3 (30-101)	<10-3
Minimal nocturnal saturation (%)	76.8±11	81.6±7.7	72.4±3	<10-3
Mean nocturnal saturation (%)	93.2±2.6	94.3±1.5	92.1±3	<10-3

BMI: body mass index, NC: the neck circumference, AHI: apnea-hypopnea index.

**Conventional echocardiographic parameters:** details of conventional and tissue Doppler echocardiographic parameters for both groups are charted in Table 2. The comparison of the echocardiographic findings showed a higher diameter of the LVED and the LVED in patients with severe OSA compared to those with mild/moderate OSA without a statistically significant difference between the two groups. Furthermore, there was no significant difference in LVEF, FS and IVS thickness between the two groups. However, the left ventricular (LV) diastolic dysfunction assessed by E/A ratio<1 was shown in 79.6% (n=74). Patients with severe OSA had a larger RVID and a higher sPAP than the patients with mild to moderate OSA. Pulmonary hypertension was observed in 14 patients (29.2%) with severe OSA and 10 patients (22.2%) with mild to moderate OSA (Table 2).

**Table 2:** echocardiographic parameters of the study population

Echocardiographic parameters	Study population (n=93)	Mild/ moderate OSA (n= 45)	Severe OSA (n=48)	P
LVEDD (mm)	46.9±6	46.2±4.7	47.6±7.2	0.36
LVESD (mm)	29.4 ± v4.8	28.9±4.5	31.3±6.2	0.14
LVEF (%)	62.7±13.4	66.1±6.9	62.1±9.7	0.06
FS (%)	34.4±6.4	36.5±6	32.5±6.3	0.07
IVSD (mm)	12.2±2.5	11.7±2.5	12.7±2.4	0.14
LV diastolic dysfunction % (n)	79.6 (74)	71.1 (n=32)	87.5 (n=42)	0.05
Deceleration time (ms)	242±85.8	260±120.9	228.4±54.3	0.45
RVID (mm)	20.4±7.1	16.9±3.1	22.9±7.9	0.05
sPAP (mmHg)	**30±7.3**	**28.2±6.1**	**33.1±7.7**	**0.03**
Pulmonary hypertension % (n)	25.8 (24)	22.2 (10)	29.2 (14)	0.09

LVEDD: left ventricle end-diastolic diameter, LVESD: left ventricle end-systolic diameter, LVEF: left ventricle ejection fraction, FS: fractional shortening, IVSD: Interventricular septum dimension, RVID: right ventricular internal diameter, sPAP: systolic pulmonary artery pressure.

**Factors associated with OSA severity:** comparison of the echocardiographic findings revealed a higher sPAP in severe OSA patients than mild to moderate patients (33.1±7.7 VS 28.2±6.1). At univariate analysis, LV diastolic dysfunction, the RVID and the sPAP were significantly associated with OSA severity, with p=0.03, p=0.05 and p=0.05 respectively. A multiple regression analysis demonstrated that the RVID was independently correlated to severe OSA (aOR 1.33, 95%CI: 0.99-1.79; p=0.05) (Table 3).

**Table 3:** multivariate regression analysis to identify predictors of the right ventricular dilatation and hypertrophy

Independent variables	Univariate analysis P value*	Multivariate analysis		
		Odds ratio	95% CI	P value
LVESD (mm)	0.14	**-**	**-**	**-**
LVEF (%)	0.06	-	-	-
FS (%)	0.07	-	-	-
IVSD (mm)	0.14	-	-	-
LV diastolic dysfunction %	0.05	**-**	**-**	**-**
RVID (mm)	0.05	1.33	[0.99-1.79]	0.05
sPAP (mmHg)	0.03	-	-	-
Pulmonary hypertension %	0.09	-	-	-

LVED: left ventricular end-diastolic diameter, LVES: left ventricular end-systolic, FS: fractional shortening, LV: left ventricular, IVSD: interventricular septum diameter, sPAP: systolic pulmonary artery pressure, CI: confidence interval.

## Discussion

The present study sought to identify subclinical effects of severe OSA on cardiac structural and functional alterations using a two-dimensional echocardiography. The key findings of this research comprise: patients with severe OSA compared with those presenting mild and moderate OSA had a higher and significant incidence of LV diastolic dysfunction, pulmonary hypertension and a larger RVID and the RVID was independently correlated to severe OSA. Several studies revealed that OSA is associated with the presence of echocardiographic abnormalities compared with controls. Those echocardiographic remodeling are related to different physiopathological mechanisms triggered by hypoxia and sleep fragmentation, involving sympathetic hyperactivity, inflammation, endothelial disfunction and oxidative stress. Comparison of conventional echocardiographic parameters in OSA patients, without any cardiovascular comorbidities, disclosed differences according to the severity of the disease. In our study echocardiographic screening showed that LVEDD and LVESD were higher in the group of severe OSA patients compared to mild to moderate OSA. However, this difference was not statistically significant between the two groups. Several studies demonstrate changes in these parameters in OSA compared to controls [11,12]. Danica *et al*. have also shown that theses diameters were higher in OSA patients with a difference statistically significant [13]. Prior studies have noted that OSA is associated with left ventricular hypertrophy, even in the absence of hypertension, obesity, and diabetes [14]. In fact, IVS thickness is reportedly higher in patients with severe OSA than in those with moderate and mild OSA. In our study, the mean IVS thickness was 12.2±2.5mm. Literature reports concerning LVEF and FS in OSA patients are controversial. In fact, some publications reported no significant differences between OSA severity and these parameters [14,15]. However, Hjaälm *et al*. conclude that the LVEF was slightly lower in moderate to severe OSA patients than in mild subjects. Another recent report demonstrates that the severity of OSA is correlated with a reduction in LVEF [16]. In our study, mild to moderate OSA patients had lower LVEF and FS than in patients with severe OSA, but the difference was not significant differences between the two groups, with p=0.06 and p=0.07 respectively.

Even the majority of the study population had a preserved systolic function, 79.6% presented a diastolic dysfunction. This finding is in agreement with those of a recent study conducted by Imai *et al*. in 2015. In fact, the authors reported that 44% of OSA patients with normal biventricular systolic function presented different degrees of diastolic function [17]. In another report, the prevalence of diastolic dysfunction was 56.8% among patients with mild OSA, it reached 69.7% in the moderate to severe OSA group (p= 0.002) [15]. In our study, 71.1% of mild to moderate OSA patients and 87.5% of severe OSA patients had diastolic dysfunction, with p= 0.05. These results may be explained by the fact that the severe OSA patients had a lower nocturnal minimum oxygen saturation than the group of mild to moderate OSA. This difference regarding diastolic dysfunction prevalence among OSA patients are not only explained by the echocardiographic parameters used in defining but also by other patient characteristics such as associated comorbidities (obesity and diabetes) [17]. The assessment of the right ventricular chambers in our study found a higher RVID in the group of severe OSA patients rather in mild to moderate OSA subjects. The difference was statistically significant, p=0.05. In literature, accurate evaluation of the right ventricular morphology and function remains challenging in clinical practice, with conflicting results between the studies. Several studies using conventional echocardiography and tissue doppler imaging demonstrated that OSA patients present frequently structural and functional alterations of the RV [18,19] and a correlation between these parameters and the severity of OSA [20]. In contrast, other studies did not reveal any change in the RV morphology and structure in OSA patients [21,22].

The prevalence of pulmonary hypertension in OSA patients ranges between 12 and 70%, according to the severity of OSA, the sPAP evaluation method and time of measurement. Pulmonary hypertension is often associated with chronic obstructive pulmonary disease and cardiac comorbidities including hypertension and left ventricular dysfunction [23]. However, numerous studies showed that permanent pulmonary hypertension may also develop in OSA patients without any known cardiopulmonary disorders [24] and patients with moderate to severe OSA had higher sPAP than healthy controls [25,26]. The results of our study were in accordance with those findings. In fact, 25.8% of the study population had a pulmonary hypertension with a mean sPAP equal to 30±7.3mmHg. On the other hand, the mean value of sPAP was higher in severe OSA patients than in mild to moderate OSA subjects. The difference was statistically significant between the two groups (p=0.03). According to those findings, severe OSA is associated to a higher incidence of echocardiographic abnormalities. Furthermore, multivariable regression analysis revealed that the RVID was shown independently associated with severe OSA (aOR=1.33), in this study.

An independent effect of OSA on RV structure and performance was observed also in a number of studies, showing a correlation between AHI and RV parameters, such as RVID [27], right ventricular wall thickness (RVWT) and tricuspid annular systolic excursion (TAPSE). According to those studies, various mechanisms could lead to RV dysfunction in patients with OSA such as increased venous return and volume overload of the right ventricle during apnea periods and pulmonary hypertension [28,29]. While, this study included 93 OSA patients without any cardio-pulmonary comorbidities, it has several limitations. First, the assessment of echocardiographic parameters was performed by different practitioners which represents the main limitation of the study. However, all those practitioners have no idea about the severity of the OSA. The second limitation consists on the lack of the quantitative evaluation of some echocardiographic parameters, such as TAPSE, which are determined by eyeballing process. Finally, the RV remodeling was shown an independently associated to the severity of OSA using a standard bidimensional echography. Recently, new advanced technologies such as Speckle Tracking Echocardiography (STE) and real-time 3D echocardiography can provide more accurate assessment of the RV function and structure.

## Conclusion

In our study, the assessment of echocardiographic parameters highlights a higher incidence of LV diastolic dysfunction, higher RVID and sPAP among severe OSA patients. The RVID was shown independently associated to the severity of the OSA. Moreover, there is the need for further study regarding RV involvement, as current data yields conflicting results.

### What is known about this topic

- Obstructive sleep apnea (OSA) is a common sleep-related disorder, implicated in many serious cardiovascular diseases including cardiac remodeling and dysfunction;
- Most of the investigations were focused on the left heart.

### What this study adds

- Conflicting results concerning the effect of OSA on RV;
- Relationship between OSA severity and echocardiographic parameters.
